# The match-play sprint performance of elite senior hurlers during competitive games

**DOI:** 10.1371/journal.pone.0215156

**Published:** 2019-04-24

**Authors:** Damien Young, Giuseppe Coratella, Shane Malone, Kieran Collins, Laurent Mourot, Marco Beato

**Affiliations:** 1 Research Unit EA3920 Prognostic Markers and Regulatory Factors of Cardiovascular Diseases and Exercise Performance, Exercise Performance Health, Innovation Platform, University of Bourgogne Franche-Comté, Besançon, France; 2 Department of Biomedical Sciences for Health, University of Milan, Milan, Italy; 3 Gaelic Sports Research Centre, Institute of Technology Tallaght, Tallaght, Dublin, Ireland; 4 The Tom Reilly Building, Research Institute for Sport and Exercise Sciences, Liverpool John Moores University, Liverpool, United Kingdom; 5 Tomsk Polytechnic University, Tomsk, Russia; 6 Faculty of Health and Science, Department of Science and Technology, University of Suffolk, Ipswich, United Kingdom; Universita degli Studi di Verona, ITALY

## Abstract

The typical sprint profile in elite hurling has yet to be established. The purpose of this study was to investigate the sprinting demands of elite hurling competition and characterize the sprinting patterns of different playing positions. GPS (10-Hz, STATSports Viper) were used to collect data from 51 hurlers during 18 games. The total sprint (≥22 km·h^-1^) distance (TSD), the number of sprints (NOS) classified as length (<20 m, ≥20 m) and relative speed thresholds (<80%, 80–90%, >90%), the between-sprint duration and the number of repeated-sprint bouts (≥2 sprints in ≤60 s) were analyzed. The NOS was 22.2 ± 6.8 accumulating 415 ± 140 m TSD. The NOS <20 m, ≥20 m was 14.0 ± 4.7 and 8.1 ± 3.6 respectively. The NOS <80%, 80–90% and >90% was 10.6 ± 4.3, 8.2 ± 3.6, 3.4 ± 2.4 respectively. The between-sprint duration and the repeated-sprint bouts were 208 ± 86 s and 4.5 ± 2.6 respectively. TSD (ES = -0.20), NOS (ES = -0.34), NOS <20 m (ES = -0.33), ≥20 m (ES = -0.24), 80–90% (ES = -0.35) >90% (ES = -0.13) and repeated-sprint bouts (ES = -0.28) decreased between-halves. Full-backs performed a lower NOS <80% than half-backs (ES = -0.66) and a shorter mean duration of sprints than half-backs (ES = -0.75), midfielders (ES = -1.00) and full-forwards (ES = -0.59). These findings provide a sprint profile of elite hurling match-play that coaches should consider to replicate the sprint demands of competition in training.

## Introduction

Hurling is a field-based stick and ball invasion-type team sport native to Ireland, which is played between two opposing teams of 15 players. The aim of the game is to outscore the opposition by striking the ball between the opposition’s goal posts [1], over the crossbar (1 point) or between and under the crossbar (3 points) [2,3]. The playing positions consist of 1 goalkeeper and 14 outfield players (full-backs, half-backs, midfielders, half-forwards, and full-forwards) who compete on a playing pitch which is 140 m long and 88 m wide over a duration of 70 minutes (min) (two 35-min halves) [2,3]. In each positional line, there is a convention of player-to-player marking, where the attackers’ role is to invade the defenders’ area and score. The defenders are tasked with preventing the attackers from scoring, while the midfielders act as a link between attack and defense [1,2]. Elite senior hurlers compete for National hurling League, Provincial and All-Ireland championships [1].

The use of global positioning satellite (GPS) technology has facilitated the collection of distances covered across low- and high- intensity efforts [2,4–12]. Total distance (TD), relative speed, high-speed running (HSR), sprint distance, peak speed were reported at senior [9,10] and U21 level [2] using GPS. Elite senior hurlers [10] cover similar relative TD to elite U21’s [2] but cover higher relative TD than sub-elite senior hurlers [9]. However, comparable peak speeds and total sprint distance have been found between senior (elite and sub-elite) [9,10] and U21 hurlers [2]. Positional differences in the match-play running performances have been found in hurling [2,10,13,14], like in other team sports [15–17]. Differences in TD and HSR were found between positions in hurlers, with midfielders in senior [10] and half-backs, midfielders and half-forwards in U21 undertaking the highest running performances (TD and HSR) [9]. Importantly, running performance decrements occur in the second half in hurling [2,9,10], similar to other team sports, which are also shown to be position specific [15,16,18].

In addition to the running metrics previously reported, the distance covered over 22 km·h^-1^ was identified as sprint distance in hurling [2,10]. Although previous research in senior hurling has provided important information about the match-play running demands, details which describe the sprint profile of players is limited to total sprint distance [10] and relative entries sprinting [9]. No research to date in senior hurling has provided information about the specific sprint demands of competitive match-play. Hurlers’ total sprint distance was found to decrease in the second half and to be position specific in both senior [2] and U21 [10] hurlers. However, it has been proposed that a focus only on total sprint distance does not provide sufficient information about the physical demands in team sport due to the intermittent nature of match-play [19]. Indeed, while the number of sprints and mean length of sprint between halves and positions are reported in U21 hurling [2], they are unknown in senior hurling. Additionally, given the dynamic nature of team invasion games, players may have to reproduce peak speed or near-to-peak speed sprints over various distances interspersed with various recovery periods [19]. Consequently, an in-depth analysis of the sprint demands in hurling should consider the number of sprints over different distances and different durations, as assessed in soccer [19–21], Rugby League [22] and hockey [23]. In addition, describing the intensities of sprints starting from the lowest sprint threshold (22 km·h^-1^) up to the players’ peak speed would provide coaches with specific details of the very high-intensity demands of competition. In various team sports, players are required to repeat high-speed actions followed by brief recovery periods [19–23]. This capability to reproduce sprints within a given period has been termed repeated-sprint ability [20]. It has been suggested that games could potentially be decided on occasions where repeated sprinting is required [19,23]. This repeated-sprint ability has been assessed in soccer [19–21], Rugby League [22] and hockey [23] but it is yet to be described in hurling.

Currently, there is no detailed sprint analysis data available for senior hurlers, which can inform coaches about the number, the lengths and the duration of sprints and the duration between sprints, the number of repeated-sprint bouts or the range of speeds achieved during sprint efforts. In addition, no information is available about the differences in sprinting demands between halves and between playing positions. The lack of specific match-play sprint demands makes the design and application of match- and position-specific sprint training programs difficult. Therefore, the aims of this study were 1) to describe the sprint analysis of elite senior hurling players during competitive match-play, 2) to describe the differences in sprint profiles between halves of play and 3) between positions. It is hypothesized that the sprint metrics would decrease in the second half and there would be a difference between positions.

## Methods

### Experimental approach to the problem

The current observational study was designed to examine the sprint demands of elite male senior hurling match-play across halves of play and between positions. All players in the current study were competing at the highest level (Provincial and All-Ireland Senior Championship) and were selected as they were members of the county’s squad that season (2017–2018). All games (*n = 18*) took place between 14.00 and 21.00 hours during the competitive season (February–August). These games included all National Hurling League and Championship games played by the team over two seasons (2017–2018). The players were classified according to their playing position during each match. Data were only included if a player completed a full match (70-min). A total number of 182 data sets met this criteria and were include for analysis (full-backs: *n = 38*, half-backs: *n = 39*, midfielders: *n = 28*, half-forwards: *n = 39* and full-forwards: *n = 38*). GPS was used to determine sprint performance variables during elite senior hurling match-play. The players were requested to abstain from strenuous physical activity in the 24 hours before competitive matches [2].

### Subjects

Fifty-one (*n = 51*) elite male hurlers with a mean (*± SD*) age, height and body mass of 28 ± 4 years, 184 ± 6 cm, 88 ± 5 kg respectively, volunteered to participate in the study. All players were free from injury and had completed a minimum of an 8-week preseason training program. Each player had a minimum training experience of three years at elite senior level. Pre data collection all players participated in up to 3 organized field-training sessions, 2 gym-based sessions per week in the pre-season period and 2–3 field training sessions, and 1–2 gym-based sessions per week in the competitive phase of the season. After ethical approval, the subjects were informed of the purpose, procedures and potential risks involved. They were also informed that they were free to withdraw from the study at any time. Written informed consent and medical declaration were obtained from the participants in line with the procedures set by the local Institution’s Research Ethics Committee. The Institute review board University Franche Comté ethical committee CPP Est-1 approved all procedures, and the study was conducted according to the Declaration of Helsinki (1975) for studies involving human subjects.

### Procedures

Height and body mass were assessed without footwear and minimal clothing using a stadiometer and weighing scales (Seca 217, Seca Ltd., Hamburg, Germany). To determine the relative sprint thresholds between the existing sprint threshold (22 km·h^-1^) used in hurling and the highest speed, the players’ peak running speed was assessed during the familiarization session. To establish the mean peak speed, all players undertook a 40 m maximal running speed test. Electronic timing gates set at 10 m intervals (Smart Speed, Fusion Sport, Queensland, Australia) [24] were used to record the fastest 10 m split time measured to the nearest 0.01 s. The players commenced each sprint from a standing start with their front foot 0.5 m behind the first timing gate and were instructed to sprint as fast as possible over the 40 m distance. Each subject performed 3 trials separated by at least 3-min of rest [25].

The match-play sprint performances were recorded using 10-Hz GPS units and 100-Hz tri-axial accelerometer (STATSports, Viper, Northern Ireland: Firmware 2.28) [2,5–7]. The validity and reliability of these GPS units for measuring high-speed distance and peak speed in sports have been previously established [26,27]. The distance bias in the 400 m trial, 128.5 m circuit, and 20 m trial was 1.99 ± 1.81%, 2.7 ± 1.2%, and 1.26 ± 1.04%, respectively. Peak speed measured by the GPS was 26.3 ± 2.4 km^.^h^-1^, and a radar gun was 26.1 ± 2.6 km^.^h^-1^, with a bias of 1.80 ± 1.93%. The major finding of this study was that GPS did not underestimate the criterion high-speed distance during a 400-m trial, 128.5 m circuit, and 20 m trial, as well as peak speed [27]. The GPS unit (dimensions 86 mm x 33 mm x 14 mm, mass 50 g) was placed within a pouch between the player’s shoulder blades (upper thoracic-spine) in a sports vest and worn under the playing jersey. GPS activation and satellite lock were established 15-min before warm-up commencement [28]. The participants were familiarized with GPS technology during team training sessions before data collection [2].

Data collected from the GPS units included total sprint distance, the total number of sprints, the speed, the length and the duration of each sprint and the mean duration between sprints were collected [2,10]. A sprint was defined as running ≥ 22 km·h^-1^ for at least 1 s [2,10]. The duration between sprints was defined as the time (s) elapsed since the previous sprint. Therefore, the time started after the first sprint in either half [19]. GPS data was downloaded to a computer through the STATSport analysis software (STATSport Viper 1.2) to be stored and analyzed after each game. On downloading, each GPS unit was labelled as the playing position. A timestamp identified first and second half data and then manually exported into a Microsoft Excel spreadsheet (Microsoft, Redmond, USA). Further separation of the sprint metrics was carried out in Excel. A repeated-sprint bout was defined as a minimum of 2 sprints that occurred within a maximum of 60 s duration between sprints [20]. The number of sprints which occurred between the following ranges < 20 m, and ≥ 20 m were identified [22]. Each sprint was also further separated based on the players’ peak speed result, using the following speed thresholds: < 80% (starting from 22 km·h^-1^), 80–90%, > 90% of the individual peak speed. Each sprint was then placed within one of the three categories and the number of sprints was counted.

### Statistical analysis

All statistical analysis was performed using SPSS for Windows (Version 22, SPSS Inc. Chicago, IL, USA). Descriptive analysis and assumptions of normality were verified before parametric statistical analysis was used. Data are presented as mean, standard deviation (± SD) and 95% confidence intervals (CI). The analysis was performed using a two-way (position x half) mixed design (ANOVA). The dependent variables across the range of analysis were total sprint distance (m), the total number of sprints (≥ 22 km·h^-1^), the mean length of sprint, the number of sprints < 20 m, ≥ 20 m, peak speed, the speed of each sprint (< 80%, 80–90%, > 90%), the duration of each sprint and the mean duration between sprints were collected. The match periods and playing positions were independent factors. Statistical significance was set at an accepted level of α < 0.05. Standardized effect sizes (ES) with 95% CI were calculated with ≤ 0.2, 0.21–0.6, 0.61–1.20, 1.21–2.00 and 2.01–4.0 and interpreted as follows; *trivial*, *small*, *moderate*, *large* and *very large* differences, respectively as recommended by Hopkins [29].

## Results

The descriptive statistics for total sprint distance, peak speed, the total number of sprints, the number of sprints per distance- and speed-category, the mean length of sprint, mean sprint duration, the duration between sprints and the number of repeated-sprint bouts for the total game and per half are presented in Table 1. The players’ mean peak speed recorded in the 40 m sprint test was 31.5 ± 1.5 km·h^-1^. The total sprint distance accounted for 5% of the overall TD covered during games. Senior hurlers’ length of sprint ranged from the shortest distance of 7 m to the longest distance 33 m.

**Table 1:** The sprint analysis for the total game, first and second halves are reported. Data are presented as mean ± SD, mean difference (95% CI) and effect size.

	**Total**	**1**^**st**^ **Half**	**2**^**nd**^ **Half**	**Difference** **95% CI**	**Effect Size**
Total Sprint Distance (m)	415 ± 140	216 ± 85	199 ± 83 *	-19 (-34 to -4)	-0.20
Peak Speed (km·h^-1^)	29.9 ± 1.5	29.2 ± 1.6	29.1 ± 1.9	-0.2 (1.0 to 0.1)	-0.06
Number of Sprints (n)	22.2 ± 6.8	11.8 ± 4.2	10.4 ± 4.0 *	-1.4 (-2.1 to -0.7)	-0.34
Number of Sprints < 20 m (n)	14.0 ± 4.7	7.5 ± 3.1	6.5 ± 2.9	-1.1 (-1.7 to -0.4)	-0.33
Number of Sprints ≥ 20 m (n)	8.1 ± 3.6	4.4 ± 2.1	3.9 ± 2.1 *	-0.5 (-0.9 to -0.1)	-0.24
Mean Length of Sprint (m)	18.6 ± 3.1	18.1 ± 3.7	19.1 ± 4.2 *	0.9 (0.0 to 1.7)	0.25
Number of Sprints < 80% (n)	10.6 ± 4.3	5.5 ± 2.7	5.1 ± 2.6	-0.4 (-1.0 to 0.1)	-0.15
Number of Sprints 80–90% (n)	8.2 ± 3.6	4.5 ± 2.5	3.7 ± 2.1 *	-0.9 (-1.3 to -0.4)	-0.35
Number of Sprints > 90% (n)	3.4 ± 2.4	1.8 ± 1.4	1.6 ± 1.6	-0.1 (-0.4 to 0.1)	-0.13
Mean Sprint Duration (s)	3.0 ± 0.5	2.9 ± 0.5	3.1 ± 0.6 *	0.1 (0.02 to 0.26)	0.36
Mean Duration between Sprints (s)	208 ± 86	199 ± 88	216 ± 116	16 (-3 to 35)	0.17
Repeated-Sprint Bouts (n)	4.5 ± 2.6	2.5 ± 2.0	2.0 ± 1.5 *	-0.6 (-1.1 to -0.2)	-0.28

CI = confidence interval.

* Significantly different (p < 0.05) from first half

**Fig 1:**
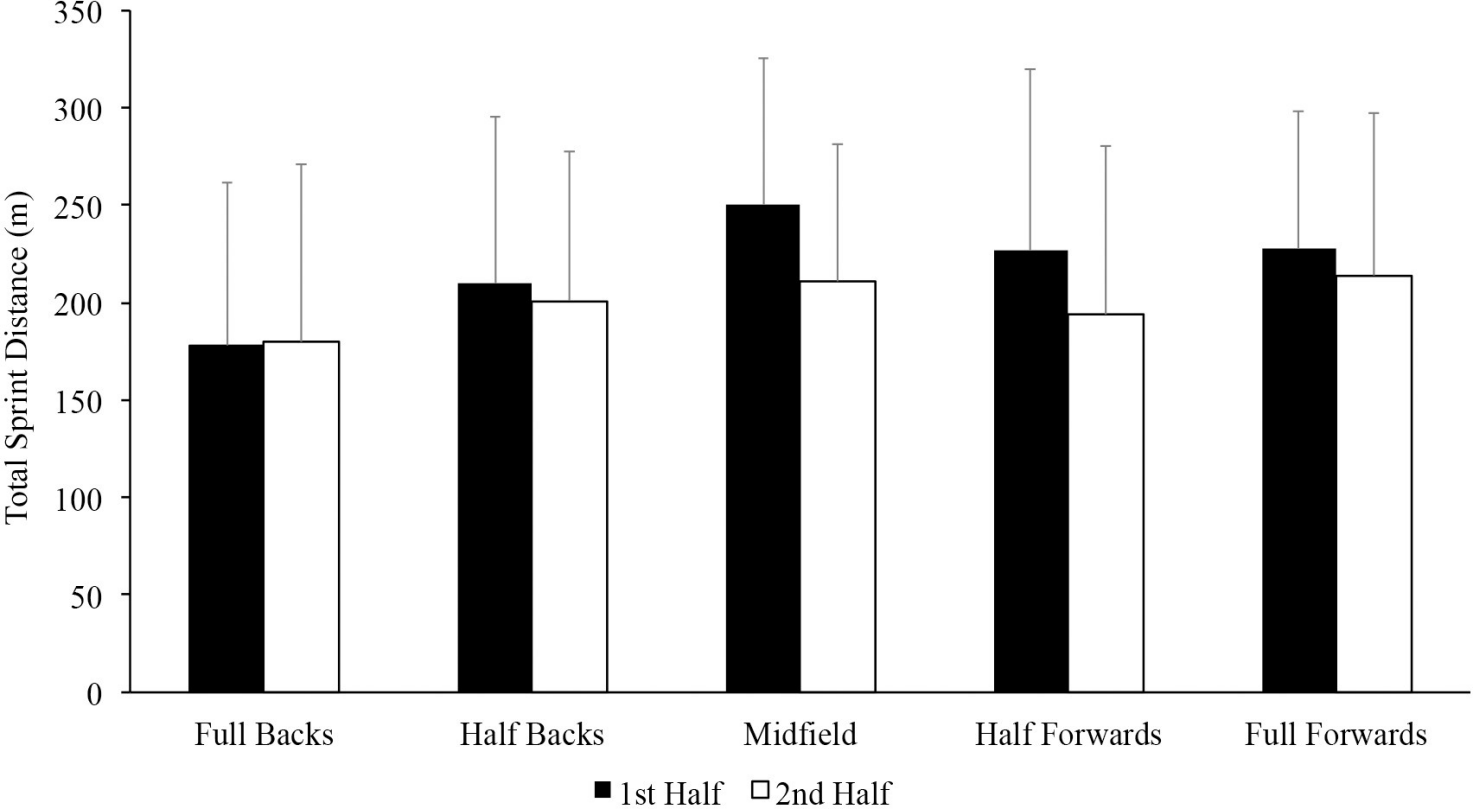
Mean (± SD) total sprint distance per position per half is presented.

The descriptive statistics for the total number of sprints and the number of sprints per distance category, the mean length of sprint, mean sprint duration and the duration between sprints per position and per half are presented in Table 2. Full backs had shorter duration of sprints compared to half backs (p < 0.05, mean difference [MD]: -0.3 95% CI -0.7 to -0.0, ES = -0.75), midfielders (p < 0.05, MD: -0.4 95% CI -0.8 to -0.1, ES = -1.00), and full forwards (p < 0.05, MD: -0.3 95% CI -0.6 to 0.0, ES = -0.59). There was no difference (p > 0.05) in any of the other speed metrics analyzed between positions (Table 1). There was no difference (p > 0.05) in the total sprint distance between full backs (357 ± 149 m), half backs (411 ± 137 m), midfielders (461 ± 110 m), half forwards (422 ± 151 m) and full forwards (442 ± 127 m). Furthermore, there was no difference (p > 0.05) in the total sprint distance per half for each position (Fig 1).

**Table 2:** The total, first and second half sprint analysis per position are reported. Data are presented as mean ± SD, mean difference (95% CI) and effect size.

		Full Backs	Half Backs	Midfield	Half Forwards	Full Forwards
Number of Sprints (n)	Total	20.5 ± 7.6	21.7 ± 6.7	23.7 ± 6.8	22.4 ± 7.3	23.2 ± 6.3
	1^st^ Half	10.3 ± 4.1	11.6 ± 4.3	12.8 ± 3.9	12.3 ± 4.4	12.3 ± 3.7
	2^nd^ Half	10.2 ± 4.6	10.2 ± 3.8	10.9 ± 3.1 *	10.1 ± 4.1 *	10.9 ± 4.1
	Diff (95% CI)	-0.1 (-1.6 to 1.4)	-1.4 (-0.1 to 3.0)	-2.0 (-4 to 0)	-2.1 (-3.8 to -0.5)	-1.4 (-3.0 to 0.2)
	ES	-0.02	-0.35	-0.54	-0.52	-0.36
Mean Length of Sprint (m)	Total	17.1 ± 2.5	18.9 ± 2.9	19.4 ± 2.8	18.8 ± 2.9	19.1 ± 3.6
	1^st^ Half	16.6 ± 3.2	18.1 ± 3.2	19.5 ± 3.2	18.7 ± 4.6	18.3 ± 3.6
	2^nd^ Half	17.6 ± 4.3	19.8 ± 4.1	19.3 ± 3.5	18.9 ± 3.4	20.0 ± 5.0
	Diff (95% CI)	0.9 (-0.8 to 2.6)	1.7 (0.0 to 3.4)	-0.2 (-2.3 to 1.9)	0.1 (-1.7 to 1.9)	1.7 (0.0 to 3.5)
	ES	0.26	0.46	-0.06	0.05	0.39
Number of Sprints < 20 m (n)	Total	13.6 ± 4.9	13.9 ± 4.7	14.5 ± 4.6	13.9 ± 4.7	14.1 ± 4.7
	1^st^ Half	6.8 ± 2.8	7.8 ± 3.3	8.0 ± 2.9	7.6 ± 3.3	7.5 ± 3.0
	2^nd^ Half	6.8 ± 3.3	6.2 ± 2.8 *	6.5 ± 2.4	6.3 ± 2.8	6.6 ± 3.1
	Diff (95% CI)	-0.6 (-4.1 to 3.0)	-1.3 (-2.9 to -0.4)	-0.8 (-1.6 to 0.0)	-0.8 (-1.5 to -0.1)	-0.7 (-1.3 to 0.0)
	ES	0.00	-0.52	-0.56	-0.42	-0.30
Number of Sprints ≥ 20 m (n)	Total	6.6 ± 3.9	7.8 ± 3.1	8.8 ± 2.8	8.7 ± 4.1	8.9 ± 3.1
	1^st^ Half	3.8 ± 2.2	3.8 ± 2.1	4.8 ± 1.8	5.0 ± 2.3	4.9 ± 1.6
	2^nd^ Half	3.3 ± 2.3	4.0 ± 2.0	4.0 ± 1.9	3.8 ± 2.5 *	4.4 ± 1.9
	Diff (95% CI)	-0.4 (-1.3 to 0.4)	0.2 (-0.6 to 1.0)	-0.8 (-1.8 to 0.2)	-1.1 (-2.1 to -0.2)	-0.5 (-1.4 to 0.4)
	ES	-0.22	0.10	-0.43	-0.50	-0.28
Mean Sprint Duration (s)	Total	2.8 ± 0.4	3.1 ± 0.4 ^a^	3.2 ± 0.4 ^a^	3.1 ± 0.4	3.1 ± 0.6 ^a^
	1^st^ Half	2.7 ± 0.5	3.0 ± 0.5	3.2 ± 0.5	3.1 ± 0.7	2.9 ± 0.5
	2^nd^ Half	2.8 ± 0.6	3.2 ± 0.6 *	3.2 ± 0.6	3.1 ± 0.5	3.2 ± 0.8 *
	Diff (95% CI)	0.1 (-0.1 to 0.4)	0.3 (0.0 to 0.5)	0.0 (-0.3 to 0.3)	0.0 (-0.2 to 0.3)	0.3 (0.0 to 0.5)
	ES	0.20	0.36	0.00	0.00	0.45
Mean Duration between Sprints (s)	Total	227 ± 100	214 ± 85	194 ± 56	221 ± 102	176 ± 60
	1^st^ Half	227 ± 105	197 ± 76	193 ± 74	197 ± 92	178 ± 81
	2^nd^ Half	226 ± 118	232 ± 128	195 ± 65	245 ± 150 *	174 ± 76
	Diff (95% CI)	-1 (-40 to 38)	36 (-3 to 74)	-2 (-47 to 50)	48 (6 to 89)	-4 (-44 to -36)
	ES	-0.01	0.33	-0.03	0.39	-0.05

Diff = Mean difference, ES = Effect size.

* Significantly different (p < 0.05) from first half.

^a^ Significantly different (p < 0.05) from full backs

**Fig 2:**
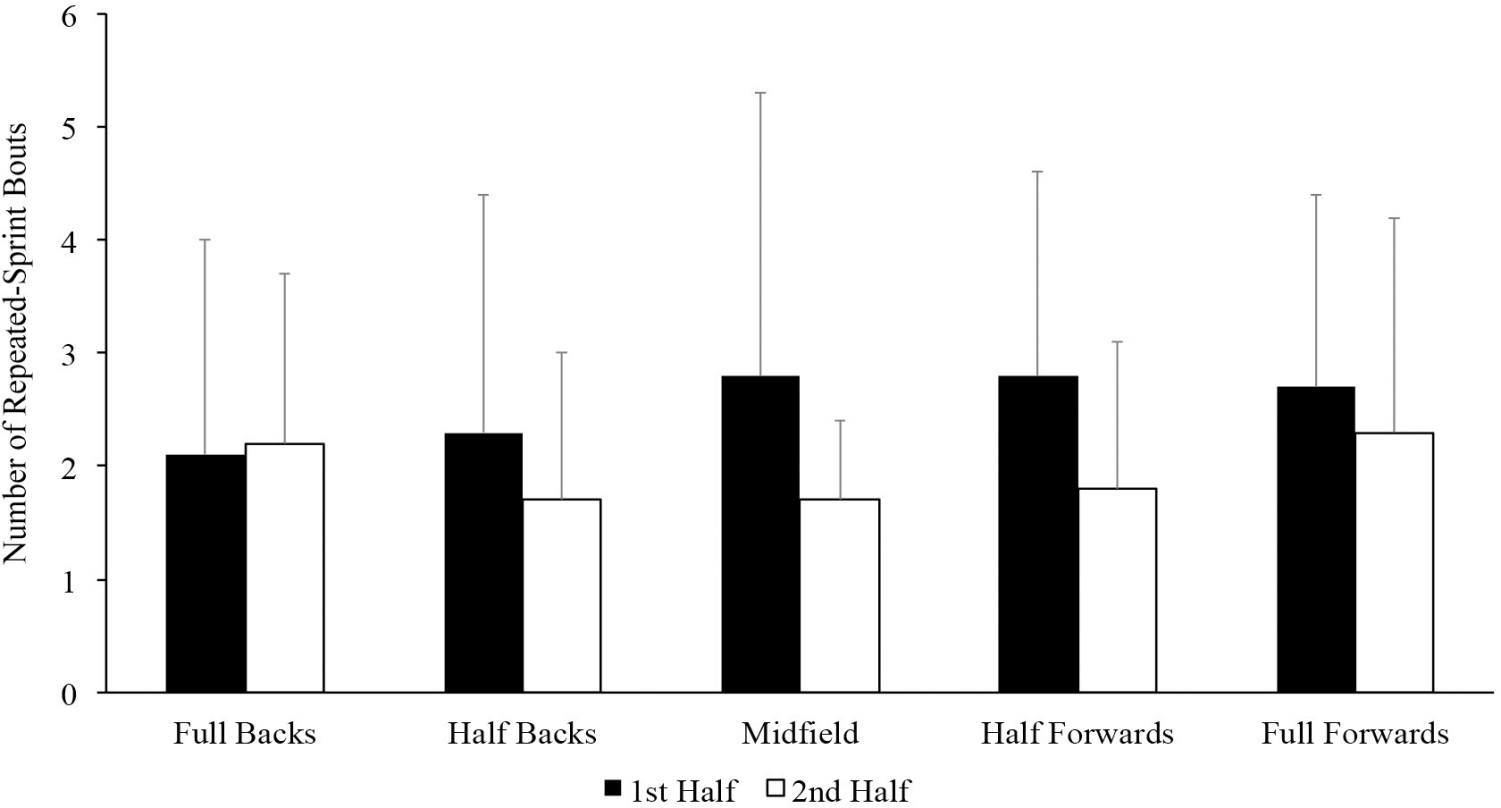
Mean (± SD) number of repeated-sprint bouts per position per half is presented.

The descriptive statistics for peak speed (km^.^h^-1^) and the number of sprints per speed intensity category and the number of repeated-sprint bouts (n) per position per half are presented in Table 3. Half backs performed a higher number of sprints < 80% compared to full backs (p < 0.05, MD: 3 95% CI 0–6, ES = 0.66). There was no difference (p > 0.05) in the peak speed (km^.^h^-1^) and the number of sprints between 80–90% and > 90% between positions. There was no difference (p > 0.05) in the number of repeated-sprint bout between full backs (4.3 ± 2.3), half backs (4.1 ± 2.6), midfielders (4.4 ± 2.6), half forwards (4.6 ± 2.6) and full forwards (5.0 ± 2.9). Furthermore, there was no difference (p > 0.05) in the number of repeated-sprint bouts per half for each position (Fig 2).

**Table 3:** The total, first and second half peak speed and number of sprints at each speed intensity category per position are reported. Data are presented as mean ± SD, mean difference (95% CI) and effect size.

		Full Backs	Half Backs	Midfield	Half Forwards	Full Forwards
Peak Speed (km·h^-1^)	Total	29.6 ± 1.4	29.5 ± 1.1	30.3 ± 1.9	29.9 ± 1.4	30.4 ± 1.8
	1^st^ Half	29.2 ± 1.5	28.7 ± 1.5	29.7 ± 1.9	29.2 ± 1.5	29.5 ± 1.8
	2^nd^ Half	28.9 ± 1.8	28.8 ± 1.2	28.8 ± 2.5 *	29.1 ± 1.8	29.8 ± 2.1
	Diff (95% CI)	-0.3 (-0.9 to 0.3)	0.1 (-0.5 to 0.7)	-0.9 (-1.8 to -0.2)	-0.1 (-0.7 to 0.6)	0.2 (-0.4 to 0.9)
	ES	-0.18	0.07	-0.41	-0.06	0.15
Number of Sprints < 80% (n)	Total	8.9 ± 4.6	12.0 ± 4.2 ^a^	11.9 ± 4.1	11.2 ± 4.1	9.4 ± 3.8
	1^st^ Half	5.5 ± 2.7	6.2 ± 3.1	5.9 ± 2.0	6.3 ± 2.7	4.9 ± 2.7
	2^nd^ Half	5.1 ± 2.6	5.8 ± 2.7	6.0 ± 2.8	4.9 ± 2.6 *	4.5 ± 2.0
	Diff (95% CI)	-0.1 (-1.2 to 1.0)	-0.4 (-1.5 to 0.7)	0.1 (-1.3 to 1.4)	-1.4 (-2.5 to -0.2)	-0.3 (-1.4 to 0.8)
	ES	-0.15	-0.14	0.04	-0.53	-0.17
Number of Sprints 80–90% (n)	Total	7.7 ± 3.5	7.2 ± 3.6	8.9 ± 3.6	7.8 ± 3.6	9.7 ± 3.5
	1^st^ Half	3.9 ± 2.1	4.1 ± 2.8	5.2 ± 2.8	4.3 ± 2.5	5.5 ± 2.0
	2^nd^ Half	3.8 ± 2.4	3.3 ± 1.7	3.7 ± 1.5 *	3.5 ± 1.9	4.5 ± 2.3
	Diff (95% CI)	-0.2 (-1.2 to 0.8)	-0.8 (-1.8 to -0.2)	-1.6 (-2.8 to -0.3)	-0.7 (-1.9 to 0.3)	-0.9 (-2.0 to 0.1)
	ES	-0.04	-0.35	-0.67	-0.36	0.46
Number of Sprints > 90% (n)	Total	3.8 ± 3.1	2.6 ± 1.6	2.9 ± 2.2	3.5 ± 2.3	4.0 ± 2.1
	1^st^ Half	1.9 ± 1.8	1.5 ± 1.1	1.7 ± 1.3	1.7 ± 1.3	2.1 ± 1.2
	2^nd^ Half	2.0 ± 1.9	1.2 ± 1.1	1.2 ± 1.5	1.8 ± 1.7	1.9 ± 1.5
	Diff (95% CI)	0.1 (-0.5 to 0.7)	-0.3 (-0.9 to 0.3)	-0.4 (-1.2 to 0.3)	0.0 (-0.6 to 0.7)	-0.1 (-0.8 to 0.5)
	ES	0.05	-0.27	-0.36	0.07	-0.15

Diff = Mean difference, ES = Effect size.

* Significantly different (p < 0.05) from first half.

^a^ Significantly different (p < 0.05) from full backs

## Discussion

The current study aimed to describe the sprint analysis of elite male senior hurling match-play across halves of play and between positions. As hypothesized, there was a decrease in sprint analysis metrics in the second half for most but not all metrics. Even though the differences were *trivial*-to-*small*, the total sprint distance, the total number of sprints, the number of sprints < 20 m and ≥ 20 m, the number of sprints < 80% and > 90% and the repeated-sprint bouts were lower (p < 0.05) in the second half. In contrast, the mean length of sprint (*small*), the duration of sprint (*small*) and the duration between sprints (*trivial*) increased in the second half (p < 0.05). There were positional differences in the mean sprint duration during the full game. Full-backs had a shorter duration of sprints compared to half-backs, midfielders and full-forwards (p < 0.05). Furthermore, full-backs performed a lower number of sprints < 80% compared to half-backs (p < 0.05). Some positions experienced *small* decreases in the number of sprints (midfielders and half-forwards), number of sprints < 20 m (half-backs), ≥ 20 m (half-forwards), mean sprint duration (half-backs and full-forwards), the duration between sprints (half-forwards), peak speed (midfielders), the number of sprints < 80% (half-forwards) and between 80–90% (midfielders) in the second half compared to the first. To the best of the authors’ knowledge, the current study was the first to examine the sprint analysis across halves of play and between positional lines during elite male senior hurling match-play.

The mean total sprint distance was higher than previously reported in elite senior hurling (319 ± 129 m) [10]. The current finding is larger than found in U21 hurling (274 ± 111 m) (2), soccer (237 ± 123 m) [30] and Australian football (328 ± 164) [31] but similar to those in Gaelic football (445 ± 269 m). The 10-min shorter match duration at U21 level [2] may explain the smaller total sprint distance covered compared to the present result. In addition, while a similar sprint zone threshold was used in Gaelic football, a higher sprint zone (≥ 24 km·h^-1^) was used in soccer [30] and Australian football [31] studies. Therefore, the distance players covered up to 24 km·h^-1^ in soccer [30] and Australian football [31] was not counted as sprint distance unlike in the current study (≥ 22 km·h^-1^). This may explain the higher total sprint distance in this study. Lastly, the 10-Hz GPS unit has been shown to be more sensitive in capturing high-intensity movements compared to GPS units measuring at 1 to 5-Hz [24]. The difference between the GPS units used in this study (10-Hz) compared to the units (4-Hz) used in the previous study [10] may explain the lower total sprint distance recorded.

Currently, there are no data to describe the total number of sprints and the mean length of sprint performed by senior hurlers. The different sprint zone classification (≥ 22 km·h^-1^ *vs* ≥ 24 km·h^-1^) makes it difficult to compare between sports. The present findings compare favorably to the number of sprints in U21 hurling (≥ 22 km·h^-1^) (18 ± 8) [2], soccer (≥ 24 km·h^-1^) (17 ± 4) [19], but slightly lower than in Australian football (≥ 24 km·h^-1^) (22 ± 9) [31]. In contrast, senior hurlers’ mean length of sprint is slightly shorter compared to rugby (21 ± 5 m) [32], soccer (21 ± 3 m) [30] and Australian football (27 m 95% CI 24.0 to 30.9 m) [33] even if similar to those found in U21 hurling (16 ± 5 m) [2]. In addition, Australian footballers can be given periods of rest during the game. Therefore, this recovery time may help them to perform more sprints and sprint over a longer distance compared to hurlers.

The number of repeated-sprint bouts have been investigated in team sports in order to gather information on the periods with the most intense sprinting demands throughout a game [19]. Soccer [20,34] and Rugby League [22] players have been shown to perform repeated-sprints bouts during match-play. The current study investigated the number of times a repeated-sprint bout (≥ 2 sprints with ≤ 60 s between sprints) [20,34] occurs in senior hurling. Even though there are methodological differences in the definitions of repeated-sprint bouts between sports, the results from the current study show that repeated-sprint bouts rarely occur in hurling like previously found in soccer [20,34] and Rugby League [22,31]. The present results show that hurlers performed a similar number of repeated-sprint bouts compared with soccer (3 ± 3 in 45-min) [20,34] but slightly higher than in Rugby League (ranged from 0–4) [22,35]. However, a different definition for a repeated-speed bout (≥ 3 sprints in ≤ 21 s) was used in Rugby League [22,31]. Thus, this may explain the difference in the number of repeated-sprint bouts between sports. In addition, the setup of the opposition formation in Rugby League may limit the space that players can sprint into before being slowed down or tackled and brought to the ground. This may also explain the lower number of repeated-sprint bouts in Rugby League compared to the present findings.

Strength and conditioning coaches usually plan and implement speed drills by marking set distances for players to sprint to and from. Therefore, to aid the development of specific speed drills, the current study separated each sprint into one of two different distance categories (< 20 m and ≥ 20 m) [4]. The greater number of sprints were performed in the < 20 m category compared to ≥ 20 m category. Similar results were found in Rugby League, since the highest frequency of sprint efforts occurred between distances of 6–10 m (39.7%) [22]. The limited space afforded to the opposition and the physical contact nature of Rugby League where players run and are stopped or slowed down by opponents may increase the number of shorter distance sprints performed and limit those sprints in the longer distance categories compared to the current study. In soccer a greater number of sprints were performed > 10 m, as players can control the ball more efficiently due to the ball being on the ground [4]. No further comparison can be made due to the limited studies that categorized the distance of sprints.

Knowledge of the players’ peak speed during match-play provides an indication of the highest speed reached during the game. It is important to note that players must be travelling ≥ 22 km·h^-1^ for at least 1 s for a sprint to be counted and the sprint distance only accumulates from this speed threshold. The present peak speed recorded compares favorably to elite senior hurling (29.6 ± 2.2 km·h^-1^) [10], U21 hurling (29.1 ± 1.9 km·h^-1^) [2], soccer (31.9 ± 2.0 km·h^-1^) [30] and Australian football (30.2 ± 1.5 km·h^-1^) [17]. The parallels in sprinting to gain possession in these invasion-type games may account for the similar peak speeds being recorded. One of the uniqueness of this study was that the sprints were divided into speed intensity categories. The present approach is novel and since no study has investigated these sprint intensity profiles in other team sport, further comparison cannot be carried out. An inverse relationship occurred across the three speed intensity categories, given that players performed the highest number of sprints closer to the minimum speed value < 80% and performed the lowest number of sprints near their players’ mean peak speed. The current results emphasize the importance of the players’ ability to perform sprints of varying speeds during match-play, as they sprint to support a teammate in possession, to create space to receive a pass, or to chase after opponents when they are in possession. This further profiling of the intensities of these sprints and quantifying the number of times players reach near their peak speed will allow coaches to prepare players for the specific sprint intensities of competition.

Similar to other team sports [17,36], the senior hurlers in the present study experienced *trivial*-to-*small* temporal decrements in sprint performance in the second half. The total sprint distance, the total number of sprints, the number of sprints < 20 m and ≥ 20 m, between 80–90%, > 90%, the number of repeated-sprint bouts and sprint duration all decreased in the second half. The current results conflict with those found in U21 hurlers, where the total sprint distance and the number of sprints remained the same between halves [2]. The 5-min additional playing time in each half, the mandatory additional 15-min that players must take to the field before the game for the warm up and the greater total volume of running performed at senior level may explain the drop-off in sprint metrics [2,9,10] compared to U21 hurling. In addition, it has previously been shown that senior hurlers [10] perform more high-speed running than U21 players, so this additional high-intensity demand could have contributed to the lower sprint performance in seniors in the second half. Research in Australian football [17], Rugby League [35] and soccer [37] showed that high-intensity exercise during the first half or quarter affects subsequent running performance in the next half or quarter of match-play. Likewise, the high-intensity efforts in the first half in the present study may explain the *trivial*-to-*small* temporal decrements in sprint performance in the second half. To the best of the author’s knowledge no other study has assessed the difference in repeated-sprint bouts between-halves. In the present study, there was a *small* decrease in the number of repeated-sprint bouts in the second half. However, from a practical viewpoint this between-half difference was less than one repeated-sprint bout. As the number of repeated-sprint bouts occurs infrequently during both halves, it can be argued that allocating time towards recreating repeated-sprint bouts may not be warranted. Interestingly, the mean length of sprint and mean duration of sprint increased by a *small* amount in the second half. As the game progresses, it may be more difficult to break down and penetrate the opposition defense. As a result, players may have to sprint longer to carry the ball into the opposition half and to support their teammates in attack or into defense to prevent scoring opportunities. There was no difference in the players’ mean peak speed and the number of sprints < 80% between halves. The peak speed in the current study compares with that found in Australian football, where players maintained their peak speed in the last quarter compared to the first [17]. Furthermore, in invasion type games, the contest for possession may motivate the players to reach peak speed, to score or to chase back to prevent a scoring opportunity. The low number of sprints < 80% performed in the first half may allow players to reach the same values in the second half. No other study has compared the between-half difference in sprint intensities making comparisons with other sports difficult.

Interestingly, there was no difference between positions for the sprint metrics analyzed, except the mean length of sprint and the number of sprints < 80%. However, in soccer positional differences have been found in the total sprint distance covered [37]. The differences in the methods used to compare positions within each study may explain the difference between studies. In the soccer study [37], the positions were described “horizontally” (full-backs vs central defenders and wide midfielders vs central midfielders) compared to “vertically” (full-backs vs half-backs vs midfield, etc.) in hurling. Those positions playing on the wing (outside positions) in soccer completed higher total sprint distance compared to central defenders, central midfielders and attackers due to the space available to run up and down [37]. In hurling, as the ball approaches a particular location in defense or attack there can be a race for possession. This contest for possession, especially in the full-forwards and full-backs where there is player-to-player marking may explain the similar sprint metrics performed between positions. In addition, the half-backs, midfielders and half-forwards may sprint to support their teammates to gain or deny possession, to score or deny a score.

The only difference between positions occurred in the mean duration of sprints and the number of sprints < 80%. Full-backs covered a *moderately* shorter mean duration of sprint compared to half-backs, midfielders and full-forwards. If the full-backs lose the race for possession they usually revert to a defensive position keeping themselves at the goal side of the attacker to prevent the full-forwards from getting inside the full-backs, making it more difficult to score. The difference in the mean duration of sprints between full-backs and full-forwards is interesting, as full-backs role is to mark full-forwards. However, in-play the full-forwards position themselves in front of the full-backs to give themselves an advantage to gain possession before the full-backs. This extra space may allow the full-forwards to sprint for a longer duration. The contrast in positioning on the pitch between half-backs and midfielders with full-backs may explain the shorter duration of sprints between positions. Half-backs and midfielders have longer distance to travel to get back into the defense to prevent scoring chances compared to the full-backs who usually stay close to the goal. Half-backs performed more sprints < 80% than full-backs. The half-backs role in retreating towards their own goal to prevent scores and moving towards midfield to attack may explain why they accumulate more sprints. In contrast, the full-backs role is to remain close to their own goal, thus limiting the number of sprints performed.

Each position maintained the total sprint distance, the mean length of sprint and the number of sprints above 90% between halves. In addition, full-backs maintained their sprint performance in all sprint metrics in the second half compared to first half. However, there were *small* differences observed in some positions between halves in the remaining sprint metrics. Even though there were *small* differences found between halves, these amounted to a decrease of 1–2 sprints and 1 km·h^-1^ in peak speed in the second half compared to the first. Therefore, from a practical viewpoint players need to be conditioned to perform the same sprint metrics in each half. These *small* differences in the second half may be due to the total volume of running performed during the game, the match outcome, players’ fitness levels or team tactics [2,10]. Interestingly, the knock-on effect of the half-forwards performing less number of sprints and number of sprints < 20 m is that they experienced a longer duration between sprints. This additional time between sprints may have given the half-forwards more time to recover and perform higher intensity sprints compared to half-backs.

The present study comes with some acknowledged limitations. Firstly, this study only assessed the sprint analysis of senior hurlers during match-play and no attempt was made to include the technical skills of the game. Since it has been reported that the majority of high-intensity efforts occur close to the ball [1], future studies should include the technical skills along with the sprint profile to understand the impact that technical skills have on sprinting during competition. Secondly, the direction of each sprint was not included. It may be interesting to describe the directions of sprints so that agility and change of direction can be included in speed training. Future studies should include video tracking technology so that the direction of sprints can be quantified. In addition, the movement prior to the sprint was not described. Traditionally coaches get players to sprint from a standing start in training. Therefore, describing if sprints occur from a standing or rolling start and the distance performed before the player reaches the sprint threshold would further specialize sprint training. Finally, the current study did not account for the workload completed between sprints. Even though players had ~208 s between sprints, they may have being running at high-speed and covering large distances without reaching the sprint threshold. Future studies should quantify this between-sprint workload and investigate the impact it has on subsequent sprints.

## Practical applications

The present results have several important practical implications for coaches who are preparing players for the sprint demands of hurling. Firstly, given the present results coaches should focus on the sprint distance range of < 20 m where the number of sprints are most frequent. Therefore, coaches should set up activities with sufficient distance to allow players to reach sprint speeds (> 22 km·h^-1^) and then ensure that players can maintain this sprint speed for more than 10 m. With 33 m being the maximum length of sprint performed in this study, it seems illogical to practice sprint lengths excessively longer than this, as players during match-play were found to decelerate from the sprinting zone before this distance.

Secondly, the novel approach used in this study, which quantified the intensities of sprints performed in senior hurling should be considered when performing sprint training. An emphasis can be placed on speeds between > 22 km·h^-1^ and < 80% relative speed, however, players also perform sprints > 80% and reach near their peak speed several times during the game. Even though players are taking part in sprint training, coaches should exposed players to a range of high-intensity sprints. To ensure this takes place coaches should monitor the intensity of sprints during training and set up activities with enough distance that players can reach high-intensity sprint speeds.

Finally, the players sprinted near peak speed during both halves, so the development of the players’ peak speed should be trained. Traditionally, sprint training has been recommended after the warm-up. However, results from the current study showed that players are required to perform high-speeds for the full duration of match-play. Therefore, the players should undertake drills that challenge them to reach near their peak speed in sprints during and towards the end of training where players must sprint under fatigue.

In conclusion, as hypothesized, there was a decrease in the total sprint distance, the total number of sprints, the number of sprints < 20 m and ≥ 20 m, the number of sprints < 80% and > 90% and the repeated-sprint bouts sprint analysis metrics in the second half. However, the mean length of sprint (*small*), the duration of sprint (*small*) and the duration between sprints (*trivial*) increased in the second half (p < 0.05). There were positional differences in the mean sprint duration (full-backs *vs*. all other positons) and a lower number of sprints < 80% (full-backs *vs*. half-backs) during the full game. *Small* decreases were observed in the number of sprints (midfielders and half-forwards), number of sprints < 20 m (half-backs), ≥ 20 m (half-forwards), mean sprint duration (half-backs and full-forwards), the duration between sprints (half-forwards), peak speed (midfielders), the number of sprints < 80% (half-forwards) and between 80–90% (midfielders) in the second half compared to the first. This study is the first to examine the specific sprint analysis across halves of play and between positional lines during elite male senior hurling match-play. These results will provide coaches with valuable information about the match-play sprint demands so specific conditioning programmes can be developed.

## Supporting information

S1 Dataset Sprint analysis GPS data from a full elite competitive hurling game. (XLSX)
